# Knowledge, Attitudes and Practices of Women in the Southern Region of Saudi Arabia Regarding Cervical Cancer and the Pap Smear Test

**DOI:** 10.31557/APJCP.2019.20.4.1177

**Published:** 2019

**Authors:** Enas A Dhaher

**Affiliations:** *Dean of the Female Nursing Institute, Armed Forces Hospital Southern Region of Saudi Arabia. *

**Keywords:** Cervical cancer, Pap-smear test, knowledge, Saudi Arabia

## Abstract

**Background:**

The main barrier for women to receive Papanicolaou (Pap) smear tests and immunization is lack of knowledge about the disease’s signs and symptoms, women’s attitudes toward prevention programs and cultural myths and beliefs. Therefore, the main objective of this study is to measure women’s knowledge, attitudes and practices about cervical cancer and the Pap smear test in the southern region of Saudi Arabia and to assess the findings in relation with women’s demographics.

**Methods:**

A cross sectional survey was conducted at the Armed Forces Hospital Southern Region Obstetrics and Gynecology Clinic using a self-administered questionnaire with a sample size of 255 women between the ages of 15 and 65 years.

**Results:**

Forty-three percent of the women in this region are aware of cervical cancer, but do not recognize its risk factors, implications, timing or main cause, which is Human papillomavirus (HPV). In fact, the primary source of information was obtained through social media. Only two women conducted Pap smear test and that was based on doctor’s referral, where women’s main reason from not conducting the test was feeling good and no need.

**Conclusions:**

There is a need, therefore, to create awareness programs for cervical cancer, its causes and risk factors, as well as its preventive measures for women in the southern region of Saudi Arabia.

## Introduction

Cervical cancer is the second most common cancer among women worldwide at a yearly death rate of 274,000; 88% of these cases were reported in developing countries (WHO, 2008). According to the World Health Organization (WHO, 2013) cervical cancer is highly curable, therefore, the main culprits for high mortality rates were lack of effective prevention, early detection and treatment programs, and access to available preventive programs . Epidemiological studies found that cervical cancer was highly correlated to Human papillomavirus (HPV) after controlling for other risk factors (Ferlay et al., 2015; Al Zaabie et al., 2015).

Several risk factors were studied worldwide to find that smoking, sexual relationships with multiple partners, multiple marriages, and dietary habits were leading in cervical cancer diagnoses (Hosono et al., 2010; Basu et al., 2014; Patel et al., 2018; Sharma and Pattanshely, 2018). Early marriage and childbirth and multiple pregnancies were also found to be high risk for cervical cancer (Basu et al., 2014). Muradi et al., (2017) stated that vaginal inflammation was a high risk factor for developing cervical cancer, as chronic infection could develop into cervical dysplasia and cervical cancer, in addition to poor hygiene and low socioeconomic status (Velenciuc, 2009).

Since the time of introducing the conventional Pap smear test and early detection programs, mortality rates have shown dramatic decreases in developing countries by 70% (Salslow et al., 2002). Reports from developing countries showed that the main barriers to receiving Pap smear tests were lack of overall knowledge about the disease, its signs and symptoms, the importance of prevention and the preventive measures, women’s attitudes towards the preventive measures for cervical cancer, and general myths and beliefs (Nwankwo et al., 2011; Esin et al., 2011; Reisetal, 2012). The American College of Obstetricians and Gynecologists (ACOG) urged women who had been or were still sexually active, and those at least 21-years of age to have an annual Pap smear test and pelvic examination. For those 30 years and above who have had three successive normal pelvic examinations and Pap smear tests, the ACOG advised for monitoring on longer intervals (Ozan, 2005)

According to Saudi Arabia’s cancer statistics, of the 152 cervical cancer cases detected yearly, 55 die with a 1.3 incident rate (Addar, 2017). According to Alsbeih (2014) in Saudi Arabia cervical cancer placed at the eighth common cancer among women in the age group 14 to 44. There were some studies conducted for HPV detection; 100 women were tested in Jeddah at King Fahad University Hospital resulted in a 94% negative rate for HPV (Gazzaz, 2007). Another study of volunteer, cervical cytology screening was conducted in the western region of the Kingdom with 485 women at King Abdulaziz University Hospital. Only 5.6% were found to be high-risk HPV (60 years and above), and 188 women from the 40-49 age group were found more likely to accept HPV testing (Bondagji et al, 2013). While a study in Riyadh revealed that 31.6% of 120 women had HPV 16/18, and 10 had cervical abnormality out of which six were positive HPV 16/18 (Al-Muammar et al., 2007). Al Sheikh’s (2014) study found that the HPV prevalence in the Saudi native population was still unknown. 

One recent study conducted in Riydh by Jaradi and Bwazir (2019) assessing women’s knowledge, attitudes and practices towards cervical cancer through focus group discussion with 77 women revealed that women were lacking knowledge regarding cervical cancer and pap smear test neither immunization, and screening was not necessary since they do not complain of any diseases.

The main objective of this study is to measure women’s knowledge, attitudes and practices of cervical cancer and its preventive measures in the southern region of Saudi Arabia.

## Materials and Methods

*Study design*

A cross-sectional survey of 255 Saudi women of at least 15 years of age was conducted at the Obstetrics and Gynecology Outpatient Clinic of the Armed Forces Hospitals Southern Region (AFHSR) in Saudi Arabia regardless of the visit reason. The study was conducted between March and April 2017. The clinic was selected, as it provided reproductive health care services to the entire southern region of Saudi Arabia, which included antenatal care, high-risk pregnancy, gynecology, infertility treatment, postnatal care, and family planning. Furthermore, the clinic was connected to the Obstetrics and Gynecology of the hospital where, on average, about 370 patients receive care daily.

*Study instrument*

Based on literature review self-administered questionnaire was developed, it consists of two main parts demography (15 questions) and knowledge, attitudes and practices of cervical cancer and the HPV vaccine. (15 questions). All questions were formulated in a simple direct language and the answers were given in a list of options where women supposed to choose one option only, an option was provided for the knowledge question which is” *I don’t know* “. The questionnaire was pilot tested (test-retest) with 25 women whom were not included in the study. The questionnaire was reliable with person’s correlation of 0.80. The alpha value for the overall questionnaire was 0.90. 

*Sample and data collection*

Sample size: The sample was designed assuming the total population visiting the clinic in one month was 8000 women based on clinic statistics and appointments. A sample of 250 women with a confidence level of 95% will provide 50% of women answering the questions of the entire relevant population with a 50 ± 6.1 confidence interval and 80% power to check for differences. Given the excellent response rate, the questionnaire was distributed to 275 women, and 255 questionnaires were returned with a response rate of 93%.

Data was collected daily for one month. Questionnaires with additional health education materials (healthy life style and nutritional habits) or educational materials without questionnaires were distributed in sealed envelopes to ensure randomization to all the women (simple randomization was done in that every other woman in the waiting area received a questionnaire that accounts around 50% of the attending women receive the questionnaire at the time of data collection). Women with at least 15 years of age, and all present in the clinic regardless of their reason for attendance (a client or a companion) were the target subject of the study. The researcher was available to assist illiterate women and those with questions regarding the purposes of the study.

*Ethical consideration*

This study was approved by the Research Ethical Committee at the Armed Forces Hospital in the Southern Region, prior to the research being conducted and furthermore, consent forms were signed by all participants in the research where it included the purpose of the study and ensure anonymity and privacy.

**Table 1:** Women’s Sociodemographic Characteristics (n=255)

Characteristics	n (%)
Women’s age group	
16-20	9 (3.5)
21-25	39 (15.3)
26-30	75 (29.4)
31-35	57 (22.4)
Above 35	76 (29.4)
Mean:32	
Women’s level of education	
Never went to school	27 (10.7%)
Basic schooling	41 (16.1)
Secondary Schooling	92 (36.1)
Diploma	13 (5.1)
University	82 (32.2)
Husband Educational level	
Never went to school	30 (11.8)
Basic schooling	26 (10.2)
Secondary Schooling	133 (52.2)
Diploma	10 (3.9)
University	49 (15.3)
Women’s professional career	
None	160 (62.7)
Health	7 (2.7)
Admin	19 (7.5)
Profession	20 (7.8)
Academic	49 (19.2)
Husband professional career	
None	188 (73.7)
Health	10 (3.9)
Admin	13 (5.1)
Profession	23 (9.0)
Academic	21 (8.2)
Women’s working stats	
Yes	38 (14.9)
No	217 (85.1)

**Table 2:** Marital, Obstetric and Gynecologic History of Study Population (n=255)

Characteristic	n (%)
Marriage years	
Single	5 (2.0)
Less than 10 year	129 (50.6)
10 years and more	121 (47.5)
	median=10.72 years
No. of pregnancies	
0	18 (7.1)
1-3	120 (47.1)
4-6	74 (29.0)
More than 6	43 (16.9)
	median=3.89
No. of deliveries	
0	51 (20.0)
1-3	124 (48.6)
4-6	55 (21.6)
More than 6	25 (9.8)
	median=2.95
No. of living children	
0	55 (21.6)
1-3	123 (48.2)
4-6	53 (20.8)
More than 6	24 (9.11)
	median=2.80
Ever used family planning methods	
Yes	133 (52.2)
No	122 (47.8)
Ever conduct comprehensive reproductive health assessment	
Yes	58 (22.7)
No	197 (77.3)

**Table 3:** Women’s Knowledge, Attitudes and Practices of Cervical Cancer (n=255)

Characteristics	n(%)
Ever heard of cervical cancer	
Yes	169 (66.3)
No	86 (33.7)
Sources of information	
Family/friends	22 (13.0)
Family doctor/GP	2 (1.2)
OBGYN doctor	11 (6.5)
Nurse	1 (0.6)
Media	133 (78.7)
Knowledge about risk factors for cervical cancer	
1.Early marriage	
Yes	49 (19.2)
no	50 (19.6)
Don’t know	156 (61.2)
2.Marrying more than one husband during reproductive age	
Yes	47 (18.4)
No	32 (12.5)
Don’t know	176 (69.1)
3.Close multiple pregnancies	
Yes	49 (19.2)
No	50 (19.6)
Don’t know	156 (61.2)
4.Smoking	
Yes	98 (38.4)
No	22 (8.6)
Don’t know	135 (52.9)
5.Nutritional habits	
Yes	64 (25.1)
No	46 (18.0)
Don’t know	145 (56.9)
6.Too many children	
Yes	44 (17.3)
No	62 (24.3)
Don’t know	149 (58.4)
7.Poor hygiene	
Yes	108 (42.4)
No	17 (6.7)
Don’t know	130 (51.0)
8.Low socioeconomic level	
Yes	46 (18.0)
No	50 (19.6)
Don’t know	159 (62.4)
9.Vaginal inflammation	
Yes	134 (52.5)
No	3 (1.2)
Don’t know	118 (46.3)
Number of risk factors recognized	
0	87 (34.1)
1-2	59 (23.1)
3-4	58 (22.7)
> 4	51 (20.0)
Direct cause of the cervical cancer	
HIV	1 (0.4)
HPV	0 (0.0)
Chlamydia	1 (0.4)
Don’t know	253 (99.2)
Cervical cancer can be prevented	
Yes	142 (55.7)
No	5 (2.0)
Don’t know	108 (42.4)
Ever Heard of vaccine for cervical Cancer (HPV)	
Yes	10 (3.9)
No	7 (2.7)
Don’t know	238 (93.3)
Have you ever took the vaccine	
Yes	1 (0.4 )
No	254 (99.6)

**Table 4:** Women’s Knowledge, Attitudes and Practices of Pap Smear Test (n=255)

Characteristics	n(%)
Have you heard of pap smear test	
Yes	111 (43.5)
No	144 (56.5)
Sources of information	
Family/friends	8 (7.2)
Family doctor/GP	2 (1.8)
OBGYN doctor	31 (27.9)
Nurse	3 (2.7)
Media	67 (60.4)
Pap smear can identify early asymptomatic lesions	
Yes	93 (36.5)
No	8 (3.1)
Don’t know	154 (60.4)
Early detection of cervical cancer has good effect on outcome of treatment	
Yes	142 (55.7)
No	5 (2.0)
Don’t know	108 (42.4)
If properly informed about Pap smear would you do it?	
Yes	99 (38.3)
No	76 (29.8)
Don’t know	80 (31.4)
If refuses to do Pap Smear, what are the causes?	
May be painful	11 (7.1)
I feel shy	7 (4.5)
I am healthy, no need	71 (45.5)
Husband would not agree	4 (2.6)
Physician doesn’t request	23 (14.7)
Don not know where to go	40 (25.6)
If you agree to do Pap Smear, whom do u prefer to do it?	
Family doctor	5 (5.1)
OBGYN	73 (73.7)
Private doctor	6 (6.1)
Mother and child health doctor	2 (2.0)
nurse	13 (13.1)
If you agree to do Pap smear, what is the preferred health provider gender?	
Male	5 (5.1)
Female	94 (94.9)
If you agree to do Pap smear, where is the preferred place?	
Women clinic in hospital	23 (23.1)
Obs. and Gyne in hospital	65 (65.7)
Private clinic	11 (11.1)

**Table 5:** The Relationship between Women’s Selected Characteristics and Women’s Knowledge, Attitudes, and Practices of Cervical Cancer and Cervical Cancer Prevention (n=255)

Characteristics (n)	Knowledge CCa	Knowledge Pap Smear	When properly informed about Pap smear, women would do it.	CC can be prevented
Women’s level of education				
Never went to school (27)	9 (33.3)	7 (25.9)	9 (33.3)	6 (22.2)
Basic schooling (41)	23 (56.1)	15 (36.6)	10 (24.4)	19 (46.3)
Secondary schooling (92)	64 (69.6)	39 (42.2)	30 (32.6)	53 (57.6)
Diploma (13)	6 (46.2)	7 (53.8)	9 (69.2)	9 (69.2)
University (82)	67 (81.7)	43( 52.4)	41 (50.0)	55 (67.1)
	P= 0.000*	P= 0.113	P=0.009*	P=0.010*
Women’s area of specialty				
Medical (7)	6 (85.7)	6 (85.7)	5 (71.4)	7 (100.0)
Admin (19)	16 (84.2)	12 (63.2)	9 (47.4)	11 (57.9)
Profession (23)	13 (65.0)	12 (60.0)	14 (70.0)	15 (75.0)
Academic (21)	38 (77.6)	21 (42.9)	23 (46.9)	31 (63.3)
	P= 0.049*	P=0.013*	P=0.007*	P=0.005*
No. of deliveries				
0 (51)	30 (58.8)	16 (31.4)	21 (41.2)	31 (60.8)
1-3 (124)	89 (71.8)	59 (47.6)	51 (41.1)	70 (56.5)
4-6 (55)	39 (70.9)	9 (52.7)	20 (36.4)	34 (61.8)
7 and more (25)	11 (44.0)	7 (28.0)	7 (20.0)	7 (28.0)
	P=0.029*	P= 0.041*	P= 0.780	P=0.050*

a, Cervical Cancer; *, significant at p˂0.05

*Statistical analysis*

The Predictive Analytics SoftWare 18 (PASW) was used to enter and analyze the data. Frequency distributions were used to present women’s demographic characteristics, knowledge, attitudes and practices related to cervical cancer and the Pap smear test. Cross tabulations were used to assess the demographic factor relations regarding the same.

## Results

*Sample demographic and obstetric characteristics*

The mean age of women was 32 years, with the majority above 25 years. Approximately 11% of the women were illiterate, and 32% were university graduates. The educational levels of the husbands were half-attained secondary schooling, and 19% were diploma and university degree holders. Among the women’s professional careers, 2.7% were in health professions and 19% were teachers and educators. Overall, only 15% of the women participating in the study were currently employed (Table 1).

Women’s obstetric and marital characteristics revealed half were married for more than 10 years, and the majority had four pregnancies. The median and mean number of deliveries was 2.95 and 2.80 respectively. When excluding women with no children, the mean number of children increased to 3.57. Nine percent of women reported having more than six living children, and nearly half of the women had never used family planning methods. Interestingly, only 23% of women had ever conducted a comprehensive reproductive health assessment (Table 2).

*Women’s knowledge, attitudes and practices of cervical cancer and Pap smear test.*

While close to three quarters of the women had ever heard of cervical cancer, the primary source of information was from social media, mainly Facebook and Twitter. Responding to further questions related to the risk factors of cervical cancer, around two thirds of women responded with either do not know or not a risk factor. More than half of the women recognized vaginal inflammation as a risk factor, but almost none knew the direct cause of cervical cancer. Regarding attitudes of prevention, approximately 57% responded that cervical cancer (HPV) could be prevented, only two women had ever conducted Pap- smear test based on doctors referral but close to all women had never heard of the HPV vaccine and, consequently, only one woman had received the vaccination in the United States. A new variable was created with the total number of risk factor recognized by women in four categories (0; 1-2; 3-4; > 4) to find out that the highest percentage was with those recognize no risk factors (Table 3). 

Number of risk factors was highly significant (p=0.001) with women’s level of education as those with higher education recognize more risk factors. Whereas none of the demographic factors was correlated with number of risks recognized neither the availability of cervical cancer immunization.

Regarding the Pap smear test, 43% of women had ever heard of the test through various media channels, 32% had received health and wellness information from female medical professionals, approximately 64% were unaware that the test could discover asymptomatic lesions, and 56% recognized that early detection could lead to better outcomes. Regarding attitudes towards the test, only 38% of women said they would participate in a screening if they were properly informed. The reasons mentioned for those not willing to participate ranged most from no complaints to least husband disagreement, 45.5% and 2.6% respectively. Two third of those willing to participate in the screening preferred served by a female gynecologist at an OB/GYN hospital (Table 4).

The relationship between women’s characteristics and their KAP towards Cervical cancer and Pap smear test.

Based on cross tabulation a significant relationship was found between women’s level of education and knowledge about cervical cancer (P=0.000). In addition, Women of higher education levels showed significant acceptance of participation in screening tests when properly informed (P= 0.009). Significant relationship was found between the belief that cervical cancer can be prevented with educational level (P= 0.010).

Those with medical professions or those with any other professional careers were more knowledgeable about the disease than other areas. Number of deliveries showed a significant relationship between women’s knowledge of both cervical cancer and the Pap smear test (P=0.029 and P=0.041). None of the other demographic characteristics showed a significant relationship between women’s knowledge, attitudes and practices.(Table 5).

## Discussion

In the southern region of Saudi Arabia, there were no screening programs or public educational campaigns for cervical cancer and its prevention at the time of this study. This study demonstrated that while close to two third of women recognize cervical cancer, few knew of its risk factors and almost none knew of the direct cause, which represents the studied women having less knowledge than participants in the Jeddah study and Kuwait (Sait, 2009; Al Sairafi and Mohamed , 2009 ). Previous studies found that lack of knowledge regarding risk factors of cervical cancer might be the most important factor for women not obtaining screening tests (Were et al., 2011; Aswathy et al., 2012; Nadarzynski et al., 2012). The lack of national screening programs and public education campaigns could explain why women were not aware of the disease’s risk factors. 

Regarding the Pap smear test, less than half the women were aware of it and about two thirds had no knowledge of the purpose of the test, which, again, represents a stark contrast to the Jeddah study (Sait, 2009). Additionally, half of the women thought early detection could lead to better outcomes. 

Our study showed the majority of women’s source of information regarding both cervical cancer and the Pap smear test was from social media, which is considered the most popular form of communication, including the communication of health information. Eight out of 10 internet users seek health information online, and two thirds seek social media to access health information in the United States (Child and Martin, 2012; Von Muhlen and Ohno-Machada, 2012). Social media could help improve and promote healthy communities when used properly (Ventola, 2014), increase access to educational health resources, and even track personal health progress (Lambert et al., 2012; Mac Millan, 2013; Grindrod et al., 2014). However, use of social media as the main source of health information could be dangerous in terms of content reliability and accuracy, currency, affiliation and authority (Moorhead et al., 2013; Pirraglia and Kravitz, 2012). Eventually in this study, we found that women recognized cervical cancer and the Pap smear, but were not familiar with risk factors or preventive measures. Despite the dangers of social media, it could be used as an effective educational tool in the southern region of Saudi Arabia. Well-organized media campaigns featuring famous regional figures or movie stars could be beneficial. Hyacinth et al., (2012) suggested a similar recommendation with the dissemination of information regarding knowledge and awareness of cervical cancer and the Pap smear test.

Level of education and type profession were found to be highly correlated to women’s knowledge about cervical cancer and the Pap smear test, as well as their attitudes toward preventing cervical cancer. Level of education was found to be a positive factor in other studies in Saudi Arabia and Kuwait (Amarin et al., 2008; Ravichandren et al., 2011).

Regarding attitudes toward preventive Pap Smear tests, more than half of the women agreed that early detection could prevent cervical cancer, and 38% agreed to participate in a screening program similar to the Akanb et al., (2015) and Almobarak et al., (2016) studies.

The main barrier for not getting the Pap smear test was having no complaints, which could be explained by lack of knowledge regarding the disease and prevention, as discussed above. Moreover, if women had no symptoms and did not feel sick, it was assumed that diagnostic procedures like the Pap smear test were unnecessary, the study in Riyadh supports the same result (Jaradi and Bawazir, 2019). Similar beliefs were found in studies in Indonesia, Qatar and Iran (Wong et al., 2008; Al-Ali et al., 2016; Ashtarian et al., 2017 respectively). Fear of pain and embarrassment, which could be considered a misconception of the test, were barriers found in studies in Malaysia and Ghana (Wong et al., 2008; Ebu, et al., 2015 respectively).

Another barrier mentioned by the women, no information about where to go (26.6%), considered a very important and devastating barrier, as found in the Ghana study (Ebu et al., 2015). Other barrier as waiting for the physician to recommend the Pap smear test was found in the Kuwait and Jeddah studies (Al Sairafi and Mohamed, 2009; Sait, 2009).

The aforementioned barriers could be explained as lack of awareness about cervical cancer and its prevention techniques, and lack of knowledge about the Pap smear test, and its implications and procedure. Several other studies posited the same (Asgharnia et al., 2009; Nwankwo et al., 2011; Farshbaf-Khalili et al., 2015). Our study showed that women with a higher level of education, and those with medical backgrounds or other professional careers were more likely willing to have Pap smear tests compared to those with less education, no education, or other educational backgrounds. This would support the thinking that awareness and knowledge played a significant role in women’s attitudes. Medical backgrounds and high levels education were previously found to be significant with women’s attitude of having the Pap smear test in a KAP study in The Sudan study (Almobarak et al., 2016). Conversely, among those who were willing to have the test in this study, 94% preferred a female health care provider, which was also found in a Kuwait KAP study (Al Sairafi and Mohamed, 2009), and among older American women (Guilfoyle et al., 2007). The study also found that a high number of deliveries were significantly associated with more awareness regarding cervical cancer and the Pap smear test. Which could be related to frequent contact with a medical and health staff during the reproductive life span and more chance for health education or health related information to be given.

Alsbeih (2014) concluded that neither national screening programs nor national vaccination programs against the HPV virus would be cost-effective in reducing cervical cancer in Saudi Arabia, as the reported incidence was already low. While the same study discussed the real prevalence of HPV was not recorded among native women in Saudi Arabia, our study showed that women were lacking knowledge regarding the Pap smear test, and cervical cancer risk factors, implications, and methods of prevention. Despite the recommendation from Alsbeih, women should have the right to choose screening tests, in which case, information on preventive measures need to be available to all women. Freedom of choice could be accomplished when women are well-informed of the disease and its available preventive measures. Therefore, it’s recommended in this study to organize a mass media campaign educating women about cervical cancer, the Pap smear test, and prevention measures targeted to women of the southern region of Saudi Arabia.

## Study strength and Limitations

High response rate is considered one of the study strength, variation in between the sample is also a strength but since the study is taking place at the obstetric clinic social desirability bias might be significant here.
